# Beyond Neuronal Heat Sensing: Diversity of TRPV1 Heat-Capsaicin Receptor-Channel Functions

**DOI:** 10.3389/fncel.2020.612480

**Published:** 2021-02-05

**Authors:** Yaroslav M. Shuba

**Affiliations:** Bogomoletz Institute of Physiology, National Academy of Sciences of Ukraine, Kyiv, Ukraine

**Keywords:** TRPV1, sensory neuron, smooth muscle, epithelia cells, adipocytes

## Abstract

Transient receptor potential vanilloid 1 (TRPV1) is a calcium-permeable ion channel best known for its ability to be gated by the pungent constituent of red chili pepper, capsaicin, and related chemicals from the group of vanilloids as well as by noxious heat. As such, it is mostly expressed in sensory neurons to act as a detector of painful stimuli produced by pungent chemicals and high temperatures. Its activation is also sensitized by the numerous endogenous inflammatory mediators and second messengers, making it an important determinant of nociceptive signaling. Except for such signaling, though, neuronal TRPV1 activation may influence various organ functions by promoting the release of bioactive neuropeptides from sensory fiber innervation organs. However, TRPV1 is also found outside the sensory nervous system in which its activation and function is not that straightforward. Thus, TRPV1 expression is detected in skeletal muscle; in some types of smooth muscle; in epithelial and immune cells; and in adipocytes, where it can be activated by the combination of dietary vanilloids, endovanilloids, and pro-inflammatory factors while the intracellular calcium signaling that this initiates can regulate processes as diverse as muscle constriction, cell differentiation, and carcinogenesis. The purpose of the present review is to provide a clear-cut distinction between neurogenic TRPV1 effects in various tissues consequent to its activation in sensory nerve endings and non-neurogenic TRPV1 effects due to its expression in cell types other than sensory neurons.

## Introduction

Transient receptor potential vanilloid 1 (TRPV1) is the modern designation for the long-sought hypothetical pharmacological receptor for the pungent constituent of chili peppers, capsaicin, located on pain sensory nerve endings. Capsaicin exerts multiple physiological effects (Monsereenusorn et al., 1982), but the specific ability of capsaicin (and the family of compounds known as capsaicinoids or vanilloids in general) to stimulate and then desensitize specific subpopulations of sensory nociceptor fibers, including C-polymodal nociceptors, Aδ mechano and heat nociceptors of the skin, and thin visceral afferent fibers, accompanied by the release of inflammatory neuropeptides has been known since the late 1960s (Jancsó et al., 1967). The concept of the existence of separate plasma membrane receptors for capsaicin became irrevocable after three major findings: (1) demonstration of the ability of capsaicin to open a non-specific membrane conductance pathway, resulting in a large Ca^2+^ entry in electrophysiological experimentation on rat vagal sensory neurons (Marsh et al., 1987); (2) demonstration of capsaicin’s efficacy to promote uptake of 45Ca^2+^ into a subset of neurons cultured from rat dorsal root ganglia (DRG) (James et al., 1988); and (3) demonstration of specific binding of [^3^H]RTX (resiniferatoxin, an ultrapotent capsaicin analog from *Euphorbia resinifera*) to particulate preparations from rat DRG (Szallasi and Blumberg, 1990a,b).

**FIGURE 1:**
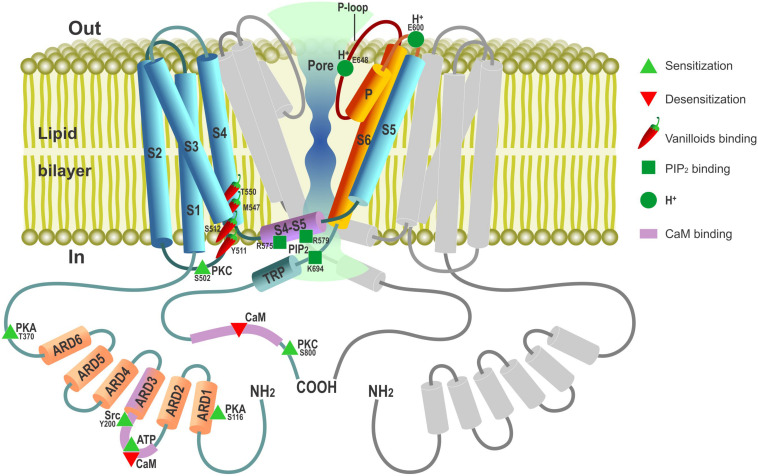
Schematic depiction of TRPV1 channel membrane topology with major functional domains. TRPV1 channel is a tetramer composed of four TRPV1 subunits arranged around a central ion-conducting aqueous pore. Only one TRPV1 subunit is shown in color (not to scale) with the second one disposed at 180° to it depicted in gray; the third (at 90°) and fourth (at 270°) subunits are omitted to open the view. Cylinders – α-helical segments, including S1…S6 transmembrane domains, P pore(P)-loop α-helix, ARD1-6 – ankyrin-repeat domains, TRP box and S4–S5 linker. Parts of TRPV1 subunit contributing to ion-conducting pore are presented in burgundy-orange. Amino acids involved in PIP2 binding are presented according to Poblete et al. (2015).

Using the strategy of expression cloning, the functional cDNA encoding a capsaicin receptor from rat DRG neurons was isolated in 1997 (Caterina et al., 1997), starting the era of molecular characterization of this important determinant of nociceptive signaling. The cloned putative subunit of the capsaicin receptor, which was termed vanilloid receptor 1 (VR1), appeared to consist of six transmembrane segments (S1…S6) (Figure 1), forming a homotetrameric capsaicin-activated non-selective cation channel with a high degree of Ca^2+^ permeability. Structurally, VR1 showed striking similarity to the members of the transient receptor potential (TRP) family ion channel subunits. In fact, it became a founding member of a whole new vanilloid (V) subfamily of TRP channels (TRPV) prompting its renaming as TRPV1 within the unified nomenclature of TRP channels, under which designation it is currently widely known and investigated. Incidentally, the cloned capsaicin receptor appeared to be also activated by increases in temperature in the noxious range (>43°C) (Caterina et al., 1997), making it also the first representative of a family of true molecular thermal sensors (Palkar et al., 2015). Activation of heterologously expressed TRPV1 by capsaicin could be potentiated by acidic pH (Caterina et al., 1997), consistent with the known synergism of protons and capsaicin action in stimulation of the pain pathways (Bevan and Geppetti, 1994). Since its cloning in 1997, the field of TRPV1 heat-capsaicin receptors became so vast and its various aspects covered in so many excellent reviews (Winter et al., 2013; Bevan et al., 2014; Earley and Brayden, 2015; Aghazadeh et al., 2017; Belvisi and Birrell, 2017) that here we only outline key milestones in TRPV1 studies, reiterate some of its basic features, and mostly focus on its non-canonical functions.

Cloning of avian and rabbit TRPV1 and exploiting the unique feature of these two species to be insensitive to capsaicin enabled mapping the structural determinants for interaction with capsaicin and other vanilloids to a small segment of aromatic and polar residues within the cytosolic S2 and S3 linker with additional residues in S4 contributing to the binding pocket (Jordt and Julius, 2002; Gavva et al., 2004; Figure 1). Although the TRPV1 channel (as well as other temperature-gated TRPs) lacks the classical, structurally defined voltage-sensing domain characteristic of voltage-gated ion channels, it is still endowed by weak intrinsic voltage-dependent activation, which becomes evident at depolarizations to very positive non-physiological membrane potentials (V_m_) (Nilius et al., 2005). In this regard, channel gating by both heat and capsaicin is explained by the shift of its voltage-dependent activation curve toward physiological V_*m*_ (Voets et al., 2004; Nilius et al., 2005): in the event of heat due to specific thermodynamics of gating charge movement and in the event of chemical agonists due to allosteric modulation.

Disruption of the *Trpv1* gene in mice deprived animals of principal *in vivo* responses thought to be linked to putative capsaicin receptor function: eliminated capsaicin and RTX sensitivity as well as greatly, though not completely, reduced behavioral responses to noxious heat stimuli, proving that TRPV1 is involved in pain and thermal nociception of the whole animal (Caterina et al., 2000). It also largely abolished proton sensitivity and heat-evoked currents in sensory neurons from TRPV1-null mice, all in all proving that TRPV1 is essential for normal thermal nociception (Caterina et al., 2000).

Electron cryo-microscopy studies on the TRPV1 ion channel structure revealed a fourfold subunit symmetry around a central ion-conducting pathway typical of voltage-gated potassium (K_v_) channels with transmembrane segments S5–S6 and the P-loop region in between forming the pore lining (Liao et al., 2013; Figure 1). Determining the TRPV1 structure trapped in different conformational states by vanilloid agonists suggested a dual gating mechanism involving two allosterically coupled areas of constriction at the outer and the inner portions of the pore (Cao et al., 2013).

In addition to the full-length TRPV1, the existence of three TRPV1 splice variants has been described, VR.5’sv, TRPV1b, and TRPV1var, of which VR.5’sv is lacking the majority of the intracellular N-terminal ankyrin repeat domains (ARDs) and TRPV1b has partial deletion of the third ARD and adjoining polypeptide sequence, whereas TRPV1var is represented only by a dramatically truncated N-terminal intracellular region and completely lacks membrane-spanning segments and the C-terminal intracellular region (Schumacher and Eilers, 2010). Through their coexpression and formation of heteromeric complexes with TRPV1, these splice variants can modulate TRPV1 activation in a dominant negative manner (Schumacher and Eilers, 2010).

## Exogenous TRPV1 Agonists and Antagonists

Except for the aforementioned capsaicin and plant toxin RTX, TRPV1 can be activated by a heterogeneous group of natural substances, including pungent (capsaicinoids) and non-pungent (capsinoids) capsaicin-related compounds from chili peppers, pungent alkaloid from black pepper and long pepper, piperine, aromatic component from clove oil, eugenol, spicy phytochemical from ginger, gingerol, non-pungent phytochemical evodiamine (Vriens et al., 2009), and animal vanillotoxins from the venom of the tarantula (Siemens et al., 2006) as well as non-pungent long-chain synthetic capsaicin analogs, olvanil, palvanil, and arvanil (Huang et al., 2013). Notably, ethanol is also considered a TRPV1 agonist as, at 0.1–3%, it can activate native and recombinant TRPV1 and sensitize its responsiveness to capsaicin, protons, and heat (by lowering the heat threshold from ∼42 to ∼34°C) (Trevisani et al., 2002).

Although the first exposure to TRPV1 activators may cause acute pain, repeated treatment actually inhibits pain perception due to TRPV1 desensitization, thereby underlying a unique form of analgesia. In fact, application of capsaicin in the form of topical ointments has been in clinical use to alleviate chronic painful conditions for decades, whereas site-specific administration of RTX is thought to be useful to treat overactive bladder via desensitization of bladder afferents and is considered as a chronic pain reliever in cancer patients (Szallasi and Sheta, 2012; Brederson et al., 2013). However, in terms of therapeutic potential as analgesics, more attention is paid to TRPV1 antagonists. The first competitive capsaicin receptor antagonist to be proposed was a synthetic benzazepine derivative, capsazepine (Dickenson and Dray, 1991; Bevan et al., 1992). It became a classical TRPV1 antagonist proven to be useful in studying TRPV1 involvement in a particular process(es) although its *in vivo* utility is limited due to poor pharmacokinetic and numerous non-specific effects. Molecular cloning of TRPV1 stimulated the search for new small-molecule antagonists. This has led to the design of new compounds with both competitive and non-competitive mechanisms of action, some of which have advanced to clinical trials as new types of analgesics (Kort and Kym, 2012; Lee et al., 2015; Aghazadeh et al., 2017).

Among the competitive TRPV1 antagonists that found broad research utility by blocking all known modes of TRPV1 activation, including capsaicin, heat, protons, anandamide, *N*-arachidonoyl dopamine (NADA), and reversing thermal and mechanical hyperalgesia in an animal model of inflammatory pain, is the cinnamide AMG 9810 [(*E*)-3-(4-*t*-butylphenyl)-*N*-(2,3-dihydrobenzo[*b*][1,4]dioxin-6-yl)acrylamide] (Gavva et al., 2005).

## Endogenous Modulators of TRPV1

As a result of various pathologic conditions, normal nociception can be disrupted leading to hyper- or hypoalgesia. To play a role in these processes, either the levels of TRPV1 expression or the extent of its activation must change. Indeed, except for capsaicin, RTX, heat acidic pH, and some other vanilloids, TRPV1 activity can be controlled by a multitude of endogenous ligands and regulatory pathways causing direct activation, sensitization, or desensitization of the channel. In most cases, endogenous factors that positively modulate TRPV1 function are those whose levels or activity increase during inflammation. Endogenous TRPV1 ligands have been termed endovanilloids (Van Der Stelt and Di Marzo, 2004).

Among the recognized endogenous TRPV1 activators are the endocannabinoids anandamide (also known as *N*-arachidonoylethanolamine, AEA) (Zygmunt et al., 1999; Smart et al., 2000) and NADA (Huang et al., 2002), some of the metabolic derivatives of arachidonic acid (AA) produced by lipoxygenases (Hwang et al., 2000) as well as polyamines (spermine, spermidine, putrescine) (Ahern et al., 2006), all of which, to various extents, have been implicated in inflammatory condition (Van Der Stelt and Di Marzo, 2004). NADA was found to be the endogenous compound most similar to capsaicin in terms of its chemical structure and the potency toward TRPV1 (Huang et al., 2002). The potency and efficacy of anandamide on TRPV1 is largely tissue- and species-dependent with its action being consistent with either a full or partial agonist, depending on such factors as receptor reserve, state of phosphorylation, voltage, temperature, and pH (Ross, 2003). 12-hydroperoxyeicosatetraenoic acid (12-HPETE), an immediate metabolic product of 12-lipoxygenase, is less potent than NADA but shares 3-D structural similarity with capsaicin (Hwang et al., 2000). Products of lipoxygenases have been implicated in mediating inflammatory nociception and are increased during inflammation. In particular, 12-HPETE is produced endogenously in sensory neurons and activates TRPV1 upon stimulation of sensory nerve endings by the inflammatory mediator and a potent pain-causing substance, bradykinin (Shin et al., 2002).

Exogenous and endogenous compounds whose activating effect on TRPV1 requires binding to the channel are regarded as true TRPV1 agonists. Except for those, however, there are also a number of regulatory factors capable of affecting channel activity by either increasing or decreasing its sensitivity to both chemical and thermal stimuli (Morales-Lázaro et al., 2013). The combination of direct and indirect mechanisms, on the one hand, can finely tune the TRPV1 activity but, on the other hand, can also convert normally innocuous stimuli into noxious signaling.

The TRPV1 channel contains multiple phosphorylation sites through which its activity can be regulated by various kinases, including protein kinase A (PKA), protein kinase C (PKC), Ca^2+^/calmodulin dependent kinase II (CaMKII), Src kinase, and Ca^2+^-dependent phosphatase calcineurin (Figure 1, reviewed in Rosenbaum and Simon, 2007; Bevan et al., 2014). PKC plays an important role in the development of hyperalgesia after tissue injury via sensitization of peripheral sensory afferents to thermal and mechanical stimuli (for review, see Velázquez et al., 2007). Peripheral sensitization involves PKC-mediated phosphorylation of TRPV1, making it much more responsive to the activating stimuli without gating the channel directly (Vellani et al., 2001; Bhave et al., 2003). As such, PKC provides the means of TRPV1 activity control via surface GPCRs and their respective agonists. One example of such control is represented by extracellular ATP, which is released from different cell types during tissue damage and initiates the sensation of pain in part due to sensitization of TRPV1 through the PKC-dependent pathway coupled to a surface metabotropic purinergic P2Y1 receptor (Tominaga et al., 2001). Other inflammatory mediators, such as bradykinin (Cesare et al., 1999), nerve growth factor (NGF) (Bonnington and McNaughton, 2003; Zhang et al., 2005), or prostaglandins E2 (PGE2) and I2 (PGI2), may also promote TRPV1 activity via their respective receptors (Moriyama et al., 2005). The results show that NGF binding to a surface TrkA receptor engages a signaling pathway in which Src kinase represents the terminal downstream element. Src kinase phosphorylates TRPV1 at a single tyrosine residue (Y200, Figure 1) to promote its trafficking to the plasma membrane, thereby underlying NGF-sensitizing action (Zhang et al., 2005).

cAMP/PKA-dependent phosphorylation potentiates TRPV1-mediated responses via direct channel phosphorylation and reducing its desensitization (Bhave et al., 2002; Mohapatra and Nau, 2005). Several candidate PKA phosphorylation sites have been identified within the TRPV1 structure with S116 and T370 representing the most functionally significant ones (Bhave et al., 2002; Mohapatra and Nau, 2005; Figure 1).

Transient receptor potential vanilloid 1 is also regulated by plasma membrane phosphoinositides. Phosphatidylinositol 4,5-bisphosphayte (PIP2), which is the substrate of phospholipase C (PLC), was initially described as a TRPV1 inhibitor whose decreased level during activation of PLC-linked G-protein-coupled receptors (GPCR) by pro-inflammatory agents, such as bradykinin, was suggested to promote TRPV1 sensitization (Chuang et al., 2001). However, subsequent studies reveal much more complex modes of phosphoinositide action involving not only PIP2, but also its precursor, phosphatidylinositol-4-phosphate (PIP), and different PLC isoforms. Most of the data support the notion that phosphoinositides are directly acting (in part through the TRPV1 proximal C-terminal region) positive rather than negative TRPV1 modulators whose depletion upon stimulation of Ca^2+^-sensitive PLCδ isoforms contributes to channel desensitization (reviewed by Rohacs, 2015). It was hypothesized that TRPV1-mediated Ca^2+^ entry *per se* can activate PLCδ to cause both PIP2 and PIP depletion, which, in turn, suppresses channel activity.

Ca^2+^-dependent mechanisms in general are key to TRPV1 desensitization with Ca^2+^ influx through the channel desensitizing it in a negative feedback manner (Koplas et al., 1997; Touska et al., 2011) via the mechanisms involving calmodulin (CaM) binding (Numazaki et al., 2003; Rosenbaum et al., 2004) or calcineurin dephosphorylation (Docherty et al., 1996; Mohapatra and Nau, 2005). TRPV1 Ca^2+^-dependent desensitization is subdivided onto two forms, depending on how it progresses: “Acute desensitization” characterizes a rapid decrease in channel activity within seconds upon first prolonged exposure to vanilloid agonists, whereas “tachyphylaxis” reflects reduction of the responsiveness to repetitive agonist exposures. Intracellular ATP or PIP2 can prevent tachyphylaxis, and CaM promotes it (Lishko et al., 2007). Structural and functional studies have shown that this occurs due to competitive interaction of the sensitizing agent, ATP, and desensitizer, CaM, with the same cytosolic ARD in the N-terminus of TRPV1 in which formation of the CaM complex with TRPV1-ARD is Ca^2+^-dependent (Lishko et al., 2007; Figure 1). Thus, ATP can sensitize the channel both directly by binding competitively with CaM to the TRPV1-ARD and indirectly by promoting replenishment of depleted PIP_2_ by lipid kinases. Long term, vanilloid-induced TRPV1 tachyphylaxis may also involve TRPV1 internalization followed by lysosomal degradation (Sanz-Salvador et al., 2012). This process requires channel activation and Ca^2+^ entry to the cell and is modulated by PKA phosphorylation of the TRPV1 cytosolic N terminal phosphorylation site (Sanz-Salvador et al., 2012).

A number of studies demonstrate TRPV1 contribution to mechanical sensitivity as well. In particular, TRPV1 is implicated in the mechanical stretch-evoked ATP release and purinergic signaling by the urothelium (Birder et al., 2002) as well as in afferent signaling of mechanical stimuli in the bladder (Gevaert et al., 2007) and gastrointestinal tract (Yu et al., 2016). However, in view of the fact that the TRPV1 channel is not considered to be mechanically gated, these effects may be indirect.

## TRPV1 Tissue Expression and Function

### TRPV1 Effects Within the Sensory Nervous System

**FIGURE 2:**
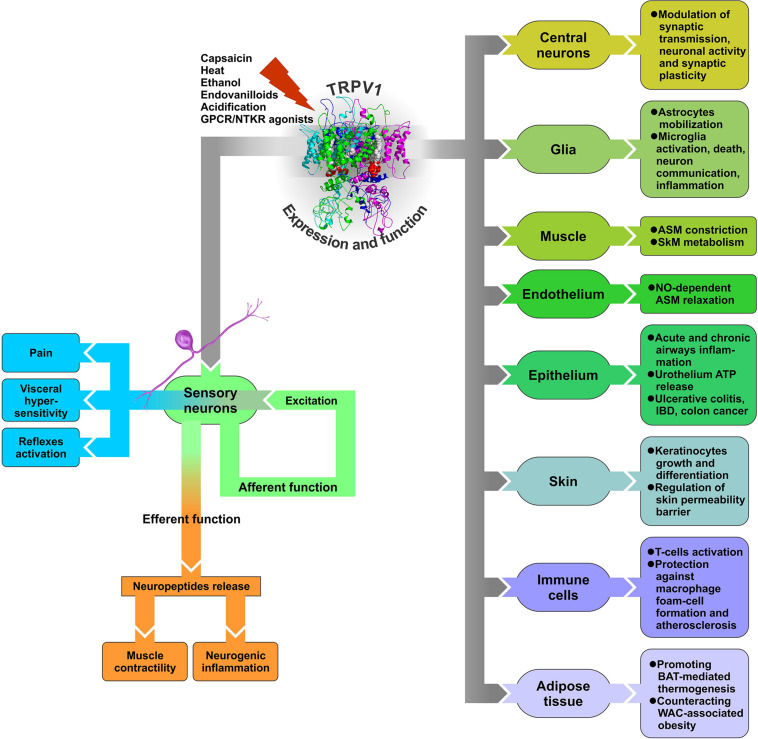
Diagram of TRPV1 expression and function in various tissues. GPCR – G protein-coupled receptor, NTKR – neurotrophic tyrosine kinase receptor, ASM – arterial smooth muscle, SkM – skeletal muscle, BAT – brown adipose tissue, WAT – white adipose tissue, IBD – inflammatory bowel disease.

With such a multiplicity of endogenous ligands and mechanisms of activation, sensitization, and desensitization, TRPV1 can operate not only as a capsaicin and heat sensor in sensory neurons involved in skin and oral sensation, but potentially participate in a plethora of other functions in cells and tissues, many of which are never exposed to exogenous irritants or ambient temperatures. Commonly, upon encountering noxious stimuli, TRPV1 channels located in sensory nerve endings open and depolarize the membrane to initiate action potentials that propagate to the central nervous system (CNS), thereby underlying sensory neurons’ “afferent function” (Figure 2). However, TRPV1 activation may also contribute to sensory neurons’ “efferent function.” Indeed, peripheral terminals of certain sensory neurons, widely distributed in skin and viscera, have the ability to release, upon stimulation with capsaicin, their transmitter content, particularly neuropeptides (e.g., Holzer, 1988; Maggi and Meli, 1988). Thus, the efferent function of these sensory neurons is realized through the effects of released mediators on neighboring cell types within the target tissue, which may have physiological or pathological significance (Figure 2).

Release of neuropeptides from capsaicin-sensitive, TRPV1-expressing sensory nerve endings as a result of their efferent function represents a key mechanism for TRPV1 involvement in physiological and pathological processes in a number of tissues, including cardiovascular, respiratory, digestive, and urogenital systems. Because this mechanism implies TRPV1 activation in sensory nerve endings with the effects of such activation on neighboring cells being indirect, mediated by neuropeptides, it can be classified as neurogenic as opposed to direct non-neurogenic effects due to TRPV1 expression in cell types other than sensory neurons.

#### Neurogenic Cardiovascular Effects

**FIGURE 3:**
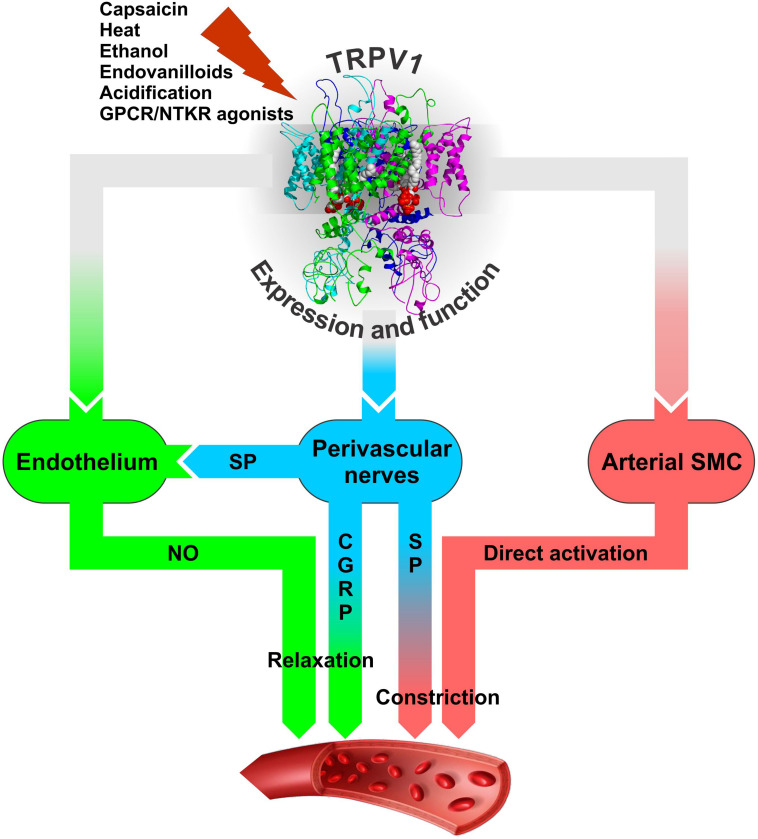
The modes of neurogenic (blue) and non-neurogenic (green and pink) TRPV1 involvement in regulation of arterial function through its expression in perivascular sensory nerves and vascular endothelial and smooth muscle cells. It is likely that the resultant effect of TRPV1 activation on vasculature and blood pressure *in vivo* would depend on a number of factors: the agonist’s nature and concentration, time of exposure (acute or chronic), route of administration (local, oral, intravenous), site of application, the presence of sensitizing or desensitizing agents that together would determine the prevalence of either constrictory or relaxant action. These factors may dynamically modulate TRPV1 activation and Ca^2+^ permeability through the phenomenon known as TRPV1 pore dilation (Chung et al., 2008) to shape TRPV1 participation in blood pressure regulation. CGRP – calcitonin gene related peptide, GPCR – G protein-coupled receptor, NTKR – neurotrophic tyrosine kinase receptor, SMC – smooth muscle cells, SP – substance P.

Transient receptor potential vanilloid 1 is densely expressed on sensory neurons innervating the myocardium, ventricles of the heart, epicardial surface of the heart, endothelial cells, and the vascular smooth muscle cells (SMC) (Szabados et al., 2020) but most likely not in cardiomyocytes [Hong et al. (2020); although see Hu et al. (2017) for a different result]. Activation of TRPV1 channels on the perivascular nerves stimulates the release of a calcitonin gene-related peptide (CGRP) and substance P to produce cardioprotection (Ustinova et al., 1995; Gazzieri et al., 2006; Zhong and Wang, 2009; Dehlin and Levick, 2014; Randhawa and Jaggi, 2017). Both CGRP and SP are known as potent vasodilating neuropeptides. For instance, TRPV1 expressed in the perivascular sensory fibers mediates the vasodilatory action of anandamide with the sequence of events involving TRPV1 activation by anandamide; release of CGRP from sensory fibers; and CGRP-mediated, endothelium-independent vasodilatation (Zygmunt et al., 1999; Figure 3). The vasodilatory action of CGRP is realized through direct action on vascular SMC to cause their hyperpolarization and relaxation through cAMP-PKA-dependent activation of K^+^ channels (Nelson et al., 1990; Earley and Brayden, 2015; Kee et al., 2018).

On the other hand, substance P can influence vascular tone in a dual manner as a vasoconstrictor or vasodilator, depending on the circumstances and the type of vessel it stimulates (Lo et al., 2018). The vascular response to substance P is primarily mediated via neurokinin type 1 receptor (NK1R), which is found on many cell types, including the smooth muscle and endothelial cells of the blood vessels. Activation of NK1R on the SMC generally causes vasoconstriction although its activation on endothelial cells induces the release of endothelium-derived vasodilators (Lo et al., 2018).

Substance P released from heart-innervating sensory nerve fibers that surround coronary vessels is a potent cardioprotector against ischemia reperfusion injury, inducing its effects via coronary vasodilation and consequent improvement of reperfusion (Ustinova et al., 1995). The facts that beneficial effects of substance P can be modulated by TRPV1-specific pharmacological agents and are impaired in TRPV1-null mice (Wang and Wang, 2005) point to a crucial role of TRPV1 in stimulating substance P release (Ustinova et al., 1995; Wang and Wang, 2005; Dehlin and Levick, 2014). Substance P, in turn, acts via endothelial NK1R to cause endothelium-dependent, NO-mediated vasodilation (Tagawa et al., 1997; Graefe and Mohiuddin, 2020; Figure 3). In addition, substance P can also provide cardioprotection by reducing ischemia/hypoxia-induced myocardial cell death by acting directly on cardiac myocytes to initiate the cell survival pathway via NK1R and AKT (Jubair et al., 2015). It should be noted, however, that except for the short-term beneficial effects in the ischemic myocardium, substance P may play a damaging long-term role in adverse myocardial remodeling and heart failure as prolonged increase in substance P can induce detrimental responses in the form of inflammation, apoptosis, matrix metalloproteinase (MMP) activation, and changes to the extracellular matrix as observed in myocarditis, volume overload, and magnesium deficiency (Dehlin and Levick, 2014).

Limb remote ischemic postconditioning (LRIP), which is known to be a safe and effective measure of cardioprotection against ischemia reperfusion injury, although still without a clear mechanism, has been shown to increase the levels of CGRP and SP in rat hearts and plasma in a TRPV1-dependent manner (Gao et al., 2015; Randhawa and Jaggi, 2017). It is concluded that LRIP engages TRPV1 to upregulate CGRP and substance P, which, in turn, limit infarct size, lower the release of myocardial infarction markers, and improve cardiac function by acting on their corresponding receptors in the heart tissue (Gao et al., 2015).

It is noteworthy that whole-body and local hyperthermia is known to induce protection against ischemia reperfusion injury of the heart by reducing the extent of infarction (Yellon et al., 1992; Gowda et al., 1998). Although this protection has been primarily attributed to the induction of heat shock proteins, the involvement of cardiac capsaicin-sensitive afferents and sensory heat-activated TRPV1 receptors in myocardial protection through the release of sensory neuropeptides cannot be excluded either.

Transient receptor potential vanilloid 1 neurogenic vascular effects have been also implicated in the pathophysiology of migraine. However, in contrast to the protective vasodilatory action of TRPV1 against ischemia reperfusion injury in the heart, CGRP release associated with neurogenic dural vasodilation is thought to be important in the generation of migraine pain. Both preclinical and clinical evidence support an important role of CGRP in migraine mechanisms (Benemei and Dussor, 2019). Besides this, a significant increase of TRPV1-immunoreactive fibers innervating scalp arteries from chronic migraine patients compared with control patients is reported (Del Fiacco et al., 2015). Activation of TRPV1-expressing trigeminal afferents by stimuli implicated in migraine (e.g., capsaicin, civamide, endovanilloids) on the one hand leads to the release of CGRP and activation of CGRP receptors on blood vessels, causing vasodilation and contributing to neurogenic inflammation and, on the other hand, to the generation of action potential signaling into the trigeminal nucleus caudalis and ultimately to headache (Benemei and Dussor, 2019).

As is mentioned above, certain arachidonate products, in particular 12-HPETE, act as TRPV1 agonists (Hwang et al., 2000). Structurally related to 12-HPETE, 20-hydroxyeicosatetraenoic acid (20-HETE), which mediates the myogenic constriction response (MCR) of small resistance arteries, also displays agonistic activity at TRPV1 (Scotland et al., 2004). MCR is an innate ability of resistance arteries to constrict in response to elevations in intraluminal pressure, which is important for maintaining peripheral resistance, organ perfusion, and systemic blood pressure. It is shown that elevation of intraluminal pressure of rat mesenteric small arteries *in vitro* is associated with generation of 20-HETE, which, in turn, activates TRPV1 on sensory vascular C-fiber nerve endings to stimulate substance P release and constriction via tachykinin NK1Rs, this time located on vascular SMCs (Scotland et al., 2004). Furthermore, agonistic action of 20-HETE on TRPV1 is enhanced under inflammatory conditions due to PKA-mediated TRPV1 sensitization and is stronger in male vs. female mice (Bubb et al., 2013). Because impairment of the 20-HETE/TRPV1 pathway may contribute to the dysregulation of organ blood flow, such as stroke or hypertension, this may, in part, explain the gender differences in susceptibility to these medical conditions.

#### Neurogenic Effects in the Respiratory, Digestive, and Urogenital Systems

Lung-innervating nociceptor sensory neurons detect noxious or harmful stimuli and, in response, initiate cough reflex, pain, and bronchoconstriction as protective measures. In the respiratory tract, TRPV1 is expressed primarily in non-myelinated sensory nerves. Activation of TRPV1-expressing nerves in the airways elicits cough, “air hunger” (dyspnea), and neurogenic inflammation (Fisher, 2009; Nassini et al., 2010). Capsaicin aerosols and high external temperature as well as endogenous inflammatory mediators, known to sensitize TRPV1, are factors that consistently promote cough. Furthermore, upregulated TRPV1 expression and function in lung afferents is characteristic of such pathologic conditions as chronic cough, asthma, and pulmonary inflammation of various etiologies (Trevisani et al., 2004; Kichko and Reeh, 2009; Choi et al., 2018; Zhang et al., 2018). In this case, the efferent function of lung afferents consists of local, TRPV1-mediated release of neuropeptides, substance P, and CGRP to activate adjacent mast cells, thereby promoting neurogenic inflammation (Figure 2; Rosa and Fantozzi, 2013).

The major cellular sources of TRPV1 in the rodent digestive tract are spinal and vagal primary afferent neurons (Holzer, 2011). Stimulation of vagal and spinal afferent nerve fibers by capsaicin, acid, and/or distension in rodents causes both local release of neuropeptide messengers (substance P or CGRP) and activation of sympathetic reflexes (Figure 2).

Acid reflux in the esophagus can induce painful sensations, such as heartburn and non-cardiac chest pain. These nociceptive symptoms are initiated by activation of TRPV1-positive afferent C fibers in the esophagus (Peles et al., 2009; Yu et al., 2014). Following esophagitis, the number of fibers responsive to capsaicin and acid increases as compared to the non-inflamed esophagus, which may contribute to esophageal hypersensitivity (Peles et al., 2009).

Transient receptor potential vanilloid 1 expression and function in the stomach, intestine, and colon afferents are responsible for mediating nociception, pain hypersensitivity, and vagal mechanosensitivity. It also participates in colorectal distension–evoked visceromotor response in the pathophysiology of ileus, irritable bowel syndrome, and inflammatory bowel disease (Yu et al., 2016). Notably, TRPV1-dependent release of substance P from primary sensory nerves in both the spinal cord (to signal pain) and the pancreas, promoting local inflammation, is considered the central feature of neurogenic pancreatitis (Vigna et al., 2014).

The digestive tract is a rich source of endocannabinoids and enzymes involved in their synthesis and metabolism. The data suggest that reciprocal changes in the TRPV1-based endovanilloid and CB1 receptor-based endocannabinoid system occur in visceral sensory fibers and that these changes could contribute to hyperalgesia and abdominal pain (Hasenoehrl et al., 2016).

In the bladder, afferent function of capsaicin-sensitive neurons consists of conveying to the CNS signals that activate the micturition reflex, cardiovascular reflexes, and perception of pain, while their efferent function provides for local regulation of muscle cell contractility, nerve excitability, blood flow, and plasma protein extravasation through the release of signaling neuropeptides, such as substance P and CGRP, from peripheral nerve endings (Maggi, 1990; Meini and Maggi, 1994).

Intravesical instillation of capsaicin in patients with hypersensitive lower urinary tract disorders attenuates their symptoms by reducing the desire to void, bladder capacity, and pressure threshold for micturition (Maggi et al., 1989). Such effects are explained by desensitization of bladder-innervating, TRPV1-expressing, capsaicin-sensitive C-fibers that detect bladder distension or the presence of irritant chemicals and, in turn, trigger reflex bladder activity and bladder pain perception. This clinical outcome substantiates the use of capsaicin and especially its ultrapotent but devoid of acute irritative action analog, RTX, to treat neurogenic bladder hyperreflexia and hypersensitivity symptoms (Cruz, 1998).

In the guinea pig bladder, substance P and CGRP released from sensory nerve endings are shown to mediate predominantly stimulatory and inhibitory action, respectively, on bladder contractility (Gillespie, 2005) although the effects of CGRP in the bladder, in contrast to those of substance P, are generally much less defined. In isolated guinea pig and rat bladders and bladder strips, capsaicin produces a TRPV1-dependent release of substance P from nerve endings with its subsequent contractile action on detrusor smooth muscle (DSM) (Maggi et al., 1991; Meini and Maggi, 1994; Philyppov et al., 2012). Moreover, a TRPV1-dependent, capsaicin-sensitive component is present in DSM contractions evoked by transmural electric field nerve stimulation (Meini and Maggi, 1994; Philyppov et al., 2012), suggesting that TRPV1 can be activated during nerve excitation independent of the presence of endovanilloids or sensitizing factors although the latter can strongly promote such activation.

### TRPV1 Expression and Function Outside Sensory Nervous System

In view of the tremendous multimodality of TRPV1 activation and regulation mechanisms, it is no surprise that, except for sensory neurons in which it acts as receptor of noxious heat, pungent chemicals, and inflammatory mediators and mediates their afferent and efferent functions, its expression is found in a multitude of other tissues and cell types. In fact, even long before TRPV1 cloning, it was known that capsaicin possesses non-specific functions that are not restricted to primary afferent neurons (Monsereenusorn et al., 1982).

It should be noted, however, that the issue of TRPV1 cellular distribution outside the sensory nervous system generates significant controversy and, in many instances, remains unresolved to date. Such situations may have a number of reasons, among which are insufficient specificity of agonists, antagonists, and regulatory factors used in functional studies; aspecific immunostaining of commercial antibodies; lack of strict correlation between the presence of mRNA and functional protein; tissue cross-contamination in RT-PCR assays; and species and age differences. In this section, we focus exclusively on direct TRPV1 effects due to its expression and function in cell types other than sensory neurons.

#### CNS

For instance, a number of brain nuclei of rats and humans is shown to express TRPV1 on mRNA and protein levels, where it is hypothesized to participate in emotion control, learning, and satiety (Mezey et al., 2000). However, the use of reporter mice expressing reporter molecules (placental alkaline phosphatase, PLAP, nuclear beta-galactosidase, nlacZ) under the control of the TRPV1 promoter (TRPV1^*PLAP–nlacZ*^ mice) enabling highly sensitive readout of TRPV1 expression patterns has shown only minimal TRPV1 expression in discrete hypothalamic regions (Cavanaugh et al., 2011). Additional *in situ* hybridization experiments demonstrate that the restricted TRPV1 expression in the brain is conserved across species (rat, monkey, human), and outside the nervous system, TRPV1 expression has been detected only in a subset of arteriolar SMC within thermoregulatory tissues, such as the cremaster muscle, dura, tongue, trachea, skin, and ear (Cavanaugh et al., 2011). Somewhat different patterns are revealed using another reporter mouse line expressing tdTomato under the control of TRPV1-Cre-mediated recombination (TRPV1-Cre:tdTomato) (Mishra et al., 2011). TRPV1 labeling was found to be almost completely restricted to sensory neurons and a subset of cells lining blood vessels and/or their precursors (Mishra et al., 2011). Surprisingly, no TRPV1 expression was detected in hypothalamus despite suggested roles of hypothalamic TRPV1 in temperature homoeostasis and in the hippocampus with very few scattered regions in the cortex (Mishra et al., 2011). Thus, even with reporter mouse lines, consistent TRPV1 labeling can be confirmed only within the sensory nervous system and some vasculature, whereas in other tissues, additional functional tests must be conducted to prove or disprove its presence. It is suggested that the reporter system may not be sensitive enough to visualize very low TRPV1 expression (Menigoz and Boudes, 2011).

Still, with respect to its role in the CNS, TRPV1, depending on its location, is believed to modulate synaptic transmission by a presynaptic mechanism as well as participate in the regulation of neuronal activity and synaptic plasticity, including LTP and LTD linked to the endocannabinoid system (Marinelli et al., 2003; Chávez et al., 2010; Grueter et al., 2010; Edwards, 2014; Saffarzadeh et al., 2015). Moreover, numerous evidence suggests TRPV1 expression and function not only in central neurons, but also in glia (Sawamura et al., 2017; Figure 2).

In CNS glia, TRPV1 is found in microglia and astrocytes (Kim et al., 2006; Miyake et al., 2015; Nam et al., 2015; Wang et al., 2019). TRPV1 activation in the substantia nigra astrocytes is shown to produce ciliary neurotrophic factor (CNTF), which prevents the active degeneration of nigral dopaminergic neurons in rat models of Parkinson’s disease (Nam et al., 2015). On the other hand, hypoxic ischemia-induced expression of astrocytic TRPV1 and the associated increase in Ca^2+^ influx promotes astrocyte migration, production, and release of pro-inflammatory cytokines (TNF, IL-1β, IL-6, and iNOS) from astrocytes into neighboring neurons to sustain epileptogenesis (Wang et al., 2019). Activation of a TRPV1-mediated increase of intracellular Ca^2+^ concentration ([Ca^2+^]_i_) accompanied by Ca^2+^-dependent cytoskeletal rearrangement and enhanced migration is also documented for retinal astrocytes in response to mechanical injury (scratch wound) (Ho et al., 2014). Overall, the data suggest that astrocytic TRPV1 is actively involved in astrocyte mobilization, which, depending on the place and context, may have a beneficial or pathologic outcome.

Microglial cells are important mediators of the immune response in the CNS. A number of studies show that TRPV1 is functionally expressed in a large proportion of microglia where, once activated, it mediates a series of transformations ranging from microglial cell death to control of microglia activation, microglia-to-neuron communication, and production and release of inflammatory mediators (Kim et al., 2006; Miyake et al., 2015; Marrone et al., 2017; Kong et al., 2019). Importantly, electrophysiological, Ca^2+^ imaging, and immunocytochemistry data indicate that microglial TRPV1 is primarily localized in mitochondrial rather than in plasma membrane (Miyake et al., 2015).

#### Bladder

Transient receptor potential vanilloid 1 cellular distribution and function in bladder tissues other than afferent nerve fibers, particularly urothelial cells, SMC, and interstitial cells, remains a matter of significant controversy with some studies presenting evidence in favor of such possibility (Birder et al., 2001; Ost et al., 2002; Lazzeri et al., 2004) and others challenging it (e.g., Everaerts et al., 2009; Yamada et al., 2009). Although the data on TRPV1 expression alone has to be taken with caution, support by functional evidence greatly increase their validity. With TRPV1^*PLAP–nlacZ*^ and TRPV1-Cre:tdTomato reporter mouse lines marked TRPV1 expression was detected in bladder sensory nerves and in arteriolar SMC but not in the urothelium (Phan et al., 2016). In arteriolar SMC, TRPV1 expression appeared age- and sex-dependent, increasing after 90 postnatal days (i.e., upon maturation) and being greater in females compared with males (Phan et al., 2016). Furthermore, in isolated arterioles from wild-type mouse bladders, capsaicin was able to induce [Ca^2+^]_i_ rise accompanied by constriction yet not in those from TRPV1-null mice (Phan et al., 2016). It was concluded that TRPV1 may be important for regulating bladder, particularly female, microcirculation.

#### Skin

Despite the results from reporter mice, the indications on extraneuronal TRPV1 expression and function continue to mount, which makes them difficult to ignore. One of the important places in which TRPV1 is detected is skin, which represents the largest organ of the body. TRPV1 is expressed in a variety of skin cells, including epidermal keratinocytes, mast cells, Langerhans cells, and sebocytes (Denda et al., 2001; Bodó et al., 2004). The data suggest that, due to its Ca^2+^ permeation and participation in Ca^2+^ signaling, TRPV1’s primary epidermal function is regulation of keratinocyte growth and differentiation (Ho and Lee, 2015). In cultured keratinocytes, TRPV1-mediated Ca^2+^ entry is shown to inhibit cell proliferation and enhance apoptosis in response to the action of endovanilloids (Tóth et al., 2011). TRPV1 is also proposed to mediate heat- and ultraviolet(UV)-induced expression of collagen-degrading matrix metalloproteinase-1 (MMP-1) in human epidermal keratinocytes (Lee et al., 2008, 2009), thereby contributing to skin aging and damage associated with these factors. Furthermore, heat and UV *per se* are capable of promoting TRPV1 protein expression in human skin *in vivo* (Lee et al., 2009).

The permeability barrier of the skin prevents transcutaneous water loss and penetration of harmful constituents from the environment. It is shown that TRPV1 activators, high temperature, and capsaicin delayed recovery of hairless mice and the human skin barrier after damage by tape stripping, and TRPV1 antagonist, capsazepine, prevented this delay (Denda et al., 2007). It was concluded that TRPV1 activation regulates the epidermal permeability barrier *in vivo*.

#### Endothelium

Transient receptor potential vanilloid 1 expression and function are also characteristic of endothelial cells (ECs) (Negri et al., 2020). This is evidenced in part by the observations that TRPV1 mRNA expression in intact and endothelium-denuded rat mesenteries is different and that activation of vascular endothelium-localized TRPV1 of the rat mesentery by anandamide or capsaicin could elicit the increase in luminal NO (Poblete et al., 2005). Endothelial TRPV1 mRNA and protein expression is further validated by RT-PCR and immunoblotting in mesenteric arteries and isolated arterial ECs from the wild-type (WT) and TRPV1-null mice (Yang et al., 2010). In the ECs from the WT mice, acute exposure to capsaicin caused [Ca^2+^]_i_ rise, increased Ca^2+^-dependent phosphorylation of endothelial NO synthase (eNOS), and stimulated NO production, whereas WT mice chronically fed a capsaicin diet for 6 months upregulated the phosphorylated eNOS level in their mesenteric arteries. All these effects were absent in TRPV1-null mice (Yang et al., 2010). Furthermore, long-term capsaicin consumption reduced arterial pressure in adult spontaneously hypertensive rats largely due to TRPV1 activation, promotion of eNOS phosphorylation, and increased NO production and, as a result, improved endothelium-dependent relaxation. It was concluded that dietary capsaicin has a beneficial effect in reducing high blood pressure through direct stimulation of endothelial TRPV1 channels (Yang et al., 2010). The obtained data also suggests that both direct EC-mediated and indirect perivascular nerve-mediated, CGRP-dependent mechanisms can participate in the acute relaxation of mesenteric arteries by capsaicin with the first one being dependent and the second one independent of eNOS and NO (Yang et al., 2010; Figure 3).

On a systemic level, capsaicin is shown to be beneficial for increasing myocardial blood flow in WT mice (Guarini et al., 2012). This effect is dependent on both TRPV1 activation and eNOS function, is compromised in a type II diabetic mouse line, and is absent in TRPV1-null mice. Studies on isolated coronary microvessels from WT mice reveal the presence of capsaicin- and acidic pH-induced, TRPV1-dependent relaxation linked to the activation of BK-type Ca^2+^-dependent K^+^-channel (apparently on arterial myocytes), which is absent in TRPV1-null mice (Guarini et al., 2012). It was concluded that TRPV1 channels mediate coupling of myocardial blood flow to cardiac metabolism via NO-dependent and BK channel-dependent pathways (Figure 3) that are corrupted in type II diabetes due to decreased myocardial TRPV1 protein expression and perhaps impaired TRPV1 pH sensitivity.

#### Muscle

Arteriolar smooth muscle is one of the few tissues in which TRPV1 expression is well established using different techniques, including TRPV1-null and reporter mice (Kark et al., 2008; Cavanaugh et al., 2011; Czikora et al., 2012; Phan et al., 2016; Hong et al., 2020). The data suggest that TRPV1 activation by various agonists in arterial SMC leads to an increase in smooth muscle intracellular Ca^2+^ concentrations and endothelium-independent vasoconstriction (Earley and Brayden, 2015; Figure 3). However, a detailed survey of TRPV1 expression and function in various vascular tissues of rats reveals that it is not uniform and may be regulated at the level of individual blood vessels even within the same tissue (Tóth et al., 2014). In rats, TRPV1 expression is detected in most of the large arteries within the skeletal muscle, mesenteric, and skin tissues as well as in the aorta and carotid arteries, but there are striking differences at the level of small arteries. Furthermore, capsaicin could constrict skeletal muscle and carotid arteries, but has no effect on the femoral and mesenteric arteries or the aorta (Tóth et al., 2014). The reasons for the observed variability are still not understood.

RT-PCR and Western blot analysis reveal TRPV1 mRNA and protein expression also in rodent skeletal muscle (Xin et al., 2005; Lotteau et al., 2013). However, immunostaining combined with confocal imaging and functional studies of intracellular Ca^2+^ dynamics are more consistent with TRPV1 localization in the membrane of sarcoplasmic reticulum (SR) rather than in the plasma membrane (Xin et al., 2005; Lotteau et al., 2013). The data suggest that skeletal muscle TRPV1 may operate as a temperature-sensitive SR Ca^2+^ leakage channel involved in the crosstalk with the ryanodine receptor (RyR1) Ca^2+^ release channel (Xin et al., 2005; Lotteau et al., 2013). Furthermore, by comparing WT and TRPV1-null mice, it is shown that TRPV1 activation by capsaicin might improve energy metabolism and endurance capacity in skeletal muscles through Ca^2+^-dependent upregulation of peroxisome proliferator-activated receptor-γ coactivator-1α (PGC-1α), mitochondrial biogenesis, and ATP production (Luo et al., 2012). It is known that constitutive mitochondrial Ca^2+^ uptake is fundamental to maintain viable levels of oxidative phosphorylation in resting cells, and constitutive low-level IP_3_R-mediated Ca^2+^ release is essential to support such uptake (Cárdenas et al., 2010). Thus, it cannot be excluded that, in tissues lacking IP_3_R, TRPV1 can take over the role of Ca^2+^ transfer to mitochondria in maintaining their functionality.

Increased muscle activity by exercise or weight training induces activation of mammalian target of rapamycin (mTOR), a member of the phosphatidylinositol 3-kinase-related kinase (PIKK) family of serine/threonine protein kinases, which promotes protein synthesis and subsequent muscle hypertrophy. It is shown that mechanical load-induced activation of mTOR and subsequent hypertrophy of mouse plantaris muscle requires activation of SR-localized TRPV1 and TRPV1-mediated [Ca^2+^]_i_ increase (Ito et al., 2013). In this case, TRPV1 is postulated to be activated by nitric oxide and peroxynitrite (Yoshida et al., 2006; Sharopov and Shuba, 2017), whose production and signaling become upregulated in overloaded muscle (Ito et al., 2013).

#### Adipose Tissue

The primary function of adipose tissue (or body fat) is to store energy in the form of fat (Cinti, 2009). There are two types of adipose tissue, the “bad” white adipose tissue (WAT) and the “good” brown adipose tissue (BAT), both of which are composed of specialized fat cells known as adipocytes. White adipose cells (WAC) serve to store excess energy as lipids and oxidize these stores when required, whereas mitochondria-rich brown adipose cells (BAC) specialize in burning lipids to generate heat (Cinti, 2009). In particular, by dissipating the chemical energy in the form of heat against cold exposure, BAC induce non-shivering thermogenesis. The stimulatory effect of cold on BAC is thought to be mediated through the sympathetic nervous system in response to the activation of thermo-TRPs in the peripheral endings of sensory neurons (Saito, 2015; Uchida et al., 2017).

Dietary capsaicin and capsinoids are known to decrease body fat and increase energy expenditure and BAC-mediated thermogenesis through a neurogenic mechanism (Saito, 2015; Uchida et al., 2017). However, functional TRPV1 expression is also detected in mouse BAT *per se*, and its expression level is found to correlate with brown adipogenesis (Kida et al., 2016). This prompts us to conclude that, by inducing the TRPV1-mediated Ca^2+^ influx, capsaicin can directly modulate either brown adipogenesis or BAC activation with concomitant strengthening of BAC-mediated thermogenesis.

Transient receptor potential vanilloid 1 expression on mRNA and protein levels is also reported in a mouse 3T3-L1-preadipocyte cell line, which replicates WAC characteristics and adipose tissues of mice and humans (Zhang et al., 2007). During chemical induction of 3T3-L1 cells to differentiate into WAC, their TRPV1 expression decreased with time, whereas cell exposure to capsaicin induced Ca^2+^ influx and prevented both adipogenesis and TRPV1 downregulation. These changes contrast with those observed during brown adipogenesis (i.e., Kida et al., 2016), suggesting the differential regulation and significance of TRPV1 between brown and white adipocytes. In addition, long-term feeding of capsaicin prevented obesity in WT but not in TRPV1-null mice assigned to a high-fat diet, indicating that TRPV1 is directly involved in white adipogenesis and obesity *in vivo* (Zhang et al., 2007).

Overall, the existing data suggest that TRPV1 plays divergent roles in BAT and WAT adipogenesis to promote BAT-mediated thermogenesis and counteract WAT-associated obesity.

#### Immune Cells and Epithelia

Although store-operated calcium entry (SOCE) is undoubtedly the major mechanism for Ca^2+^ entry in immune and epithelial cells, it was discovered that CD4-positive T-lymphocytes (CD4^+^ T-cells) in their plasma membrane also contain a TRPV1 channel (Bertin et al., 2014). Moreover, TRPV1 in CD4^+^ T-cells appeared functional, contributing to T-cell receptor (TCR)-induced Ca^2+^ influx and concomitant cell activation (Bertin et al., 2014). The data suggest that TRPV1 in CD4^+^ T-cells is part of the proximal TCR signaling cascade, wherein TCR engagement induces TRPV1 tyrosine phosphorylation by lymphocyte-specific protein tyrosine kinase (Lck) to cause TRPV1 sensitization. Thus, TRPV1 acts as a non-SOCE channel that contributes to TCR-induced Ca^2+^ influx in CD4^+^ T-cells and their activation.

Transient receptor potential vanilloid 1 is also implicated in deregulation of lipid metabolism and inflammation in macrophages (Zhao et al., 2013). The accumulation of macrophage-derived foam cells in the intima and subsequent release of inflammatory cytokines from them are critical in the initiation and progression of atherosclerosis. The formation of foam cells primarily results from uncontrolled uptake of modified low-density lipoprotein (LDL) by macrophages, leading to the excessive lipoprotein-derived lipid accumulation inside cells and induction of proinflammatory mediators. Immunohistochemical staining for TRPV1 reveals positive signals confined mainly to areas of macrophages in atherosclerotic lesions of apolipoprotein E-deficient mice (ApoE^–/–^, a model of atherosclerosis-prone mice) (Zhao et al., 2013). Induction of foam cell formation from mouse bone marrow–derived macrophages by LDL treatment increased TRPV1 expression; however, activation of TRPV1-mediated Ca^2+^ influx by exposure to agonists (capsaicin or evodiamine) protected against lipid accumulation and foam-cell formation. Additionally, capsaicin or evodiamine suppressed a TNF-α-induced inflammatory response in macrophages. Thus, activation of TRPV1 by agonists plays a protective role against foam-cell formation and development of atherosclerosis (Zhao et al., 2013).

Transient receptor potential vanilloid 1 expression and function is also documented in a number of epithelial cells, including respiratory, intestinal, and urothelium. In the lung, TRPV1 expression is found in the respiratory epithelium, which forms a protective barrier against inhaled pollutants and plays a critical role in innate airway responses to environmental pathogens and airborne allergens. In human airway epithelial cells, TRPV1 is shown to regulate inflammatory cytokine production following exposure to ambient particulate pollutants and TRPV1 agonists and to promote cell death (Veronesi et al., 1999; Reilly et al., 2005). TRPV1 expression is also upregulated in the airway epithelium from patients with obstructive airway diseases characterized by chronic inflammation of the respiratory tract, such as asthma and in animal models of asthma (Belvisi and Birrell, 2017).

Except spinal and vagal primary afferent neurons, TRPV1 expression in the digestive system is found in multiple types of non-neural cells of several species, including human: submandibular gland serous acinar and ductal cells; gastrin and parietal cells of the stomach; and epithelial cells of the esophageal, gastric, and small intestinal mucosa (Holzer, 2011). However, as in the bladder, the distribution of extra-neuronal TRPV1 in the digestive system has to be taken with caution given that TRPV1 antibodies can produce false positive staining in non-neural tissues even in TRPV1-null mice (Everaerts et al., 2009). For instance, expression profiling of various TRPV family members in colonic biopsies from patients with ulcerative colitis (UC) shows statistically decreased TRPV1 expression in their mucosal epithelium in comparison to non-inflamed intestine samples from a control group of patients with no inflammatory bowel disease (IBD) (Rizopoulos et al., 2018). In view of the fact that TRPV1 is shown to play a protective role against initiation and progression of colon cancer in mouse models (Vinuesa et al., 2012) while IBD actually predisposes to colon cancer development, it is hypothesized that decreased TRPV1 expression in the epithelium of UC samples may be indicative of the diminishing protective role of TRPV1 against colon cancer with the exacerbated colon inflammation (Rizopoulos et al., 2018). However, in another study of colonic biopsies from healthy individuals and patients with active IBD (including UC as well as Crohn’s disease), TRPV1 expression in colonic epithelium and infiltrated inflammatory cells was found significantly upregulated in IBD patients vs. controls (Luo et al., 2017) with no significant correlation detected between the level of TRPV1 expression and disease severity.

Functional expression of TRPV1 is also demonstrated in several human carcinomas (i.e., malignancies that develop from epithelial cells) and cancer cell lines derived from them, including prostate cancer, breast cancer, thyroid carcinoma, urothelial cancer, and glioma (reviewed in Bujak et al., 2019) with cancer cells generally showing lower TRPV1 expression as compared with the normal cells from which they originate. Furthermore, capsaicin belongs to the group of dietary phytochemicals demonstrating anticancer activity (Clark and Lee, 2016), and its action in glioma and urothelial cancer cells is shown to induce TRPV1-mediated, Ca^2+^-dependent apoptosis (Amantini et al., 2007; Santoni et al., 2012). A similar effect to capsaicin is also demonstrated for endovanilloids (including AEA and NADA) in high-grade astrocytomas (Stock et al., 2012), suggesting that TRPV1 agonists have potential as anticancer therapeutics.

## Conclusion

The above description, which is, by far, incomplete, indicates that widespread tissue distribution of TRPV1 supports numerous seemingly unrelated cellular functions, making it an important determinant of diverse physiological processes ranging from nociception to energy metabolism. At the same time, aberrant TRPV1 expression and/or regulation can contribute to the pathogenesis of several diseases. Even already available evidence implicates TRPV1 in pathological pain (inflammatory, visceral, cancer, neuropathic), various respiratory conditions (including chronic cough, asthma), IBD, cardiovascular diseases, interstitial cystitis, urinary incontinence, pancreatitis, and migraine (White et al., 2011). In this regard, search for new TRPV1-specific agonists and antagonists remains a priority. To address existing controversies regarding tissue-specific TRPV1 expression and function, their analysis has to be preferentially performed on a single-cell level with the use of cell type markers.

## Author Contributions

The author confirms being the sole contributor of this work and has approved it for publication.

## Conflict of Interest

The author declares that the research was conducted in the absence of any commercial or financial relationships that could be construed as a potential conflict of interest.
